# Breaking the Limitations of ‘Milk’: Research Progress on New Derivatives of Propofol

**DOI:** 10.3390/molecules31173066

**Published:** 2026-08-31

**Authors:** Ling Huang, Yu Xia, Jiajia Shi, Yin Chen, Chao Hao

**Affiliations:** 1 Department of Biomedical Engineering, College of Life Science and Technology, Huazhong University of Science and Technology, Wuhan 430074, China; d202581331@hust.edu.cn; 2 Jiangsu Key Laboratory of Marine Biological Resources and Environment, Jiangsu Key Laboratory of Marine Pharmaceutical Compound Screening, School of Pharmacy, Jiangsu Ocean University, Lianyungang 222005, China; 2025221063@jou.edu.cn (Y.X.);

**Keywords:** intravenous anesthetic, general anesthetic, propofol, gamma-aminobutyric acid receptor, pain

## Abstract

Over the past several decades, intravenously administered anesthetics such as propofol have gained widespread recognition for their use in inducing and maintaining general anesthesia. As a fast-acting, short-acting anesthetic, propofol exhibits rapid onset, brief duration, excellent induction efficacy, minimal accumulation, and promotes clear-headedness and swift recovery post-anesthesia. However, its poor water solubility necessitates formulation as an emulsion, which may cause injection site pain during administration and potentially lead to allergic reactions and lipid metabolism disorders. In light of this, pharmacologists aim to optimize propofol through structural modification, seeking to develop a systemic intravenous anesthetic that mitigates or improves these side effects.

## 1. Introduction

**Figure 1:**
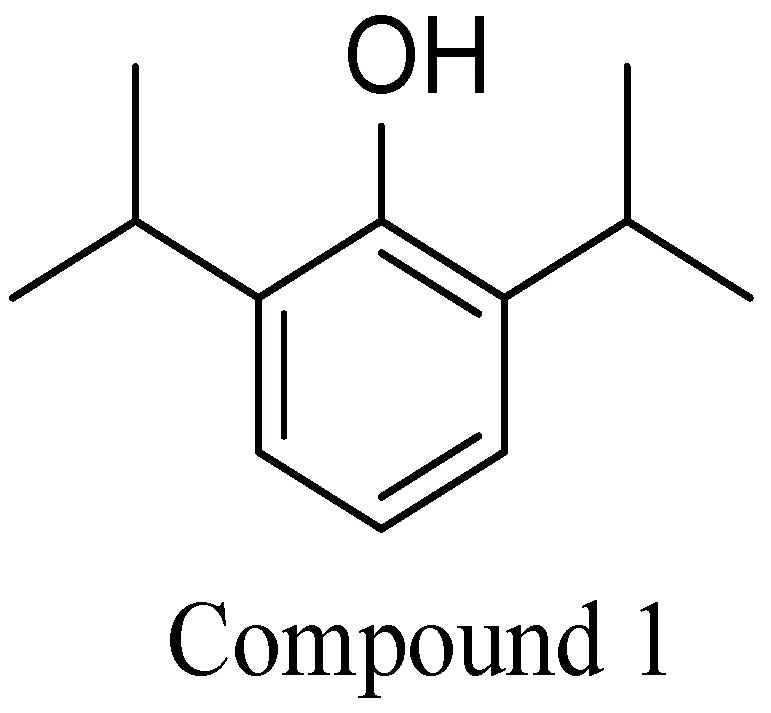
Chemical structure of propofol.

Propofol **(Compound 1)**, a general anesthetic chemically known as 2,6-diisopropylphenol (structure shown in Figure 1), belongs to the alkyl acid class of short-acting intravenous anesthetics. British veterinary scientist and Imperial Chemical Industries (ICI) researcher John B. Glen spent 13 years developing propofol. Following extensive evaluation of the anesthetic potency and pharmacokinetic properties of a series of o-alkylphenols, along with structure–activity relationship studies, he applied these findings to anesthetic development [1]. In initial clinical trials, propofol was administered as an injection dissolved in BASF’s Kolliphor EL [2]. In 1986, due to allergic reactions, this formulation was replaced by AstraZeneca’s soybean oil/propofol emulsion (branded as Diprivan), which remains in use today. Propofol primarily targets gamma-aminobutyric acid (GABA) type A receptors (GABAA receptors) and exhibits multiple mechanisms of action [3,4]. GABAA receptors belong to a class of ionotropic receptors. Upon binding with GABA molecules, chloride ions enter the neuronal cell membrane through open chloride channels. The resulting membrane hyperpolarization inhibits the generation of new action potentials [5]. Propofol can act by either enhancing GABAA receptor activity [3] or by functioning as a positive allosteric modulator of the GABAA receptor, which significantly prolongs the channel open duration and increases the opening frequency. At high doses, propofol may activate GABAA receptors even in the absence of GABA and also exhibits GABAA receptor agonist activity [6,7,8]. Within relevant concentration ranges, propofol exerts central inhibitory effects by reducing the release of neurotransmitters from presynaptic membranes in the central nervous system while increasing the levels of inhibitory neurotransmitters, thereby disrupting the efficacy of excitatory synaptic transmission [9]. Other studies indicate that the endocannabinoid system and voltage-dependent sodium channel blockade also play significant roles in activating propofol’s anesthetic effects [10].

Propofol binds to plasma proteins in vivo [11] and is inactivated through hepatic metabolism [12]. Due to its rapid distribution within tissues [13], the clinical anesthetic effect of propofol is short-lived. Following a single dose, it rapidly takes effect within 15–30 s [14]. During clinical intravenous infusion, propofol is typically inactivated within minutes after discontinuation of administration, which is particularly important for short-duration anesthetic procedures or examinations [15,16].

Anesthesia and sedation as the primary therapeutic value of propofol involve diverse domains and levels, such as induction and maintenance of anesthesia [17], anesthesia care and diagnostics [18], procedural sedation, sedation in critical care [19,20], seizure control, nausea and vomiting, and itching relief [21,22,23]. Similar to the patient-controlled analgesia (PCA) model, propofol is also used for postoperative intravenous anesthesia maintenance, with real-time dose control via infusion pumps [24]. Prior to propofol, thiopental sodium was the primary agent for inducing general anesthesia in surgical patients. Compared to propofol, thiopental sodium carries a degree of post-anesthesia side effects, such as impacts on alertness and related physical functions [25,26]. Furthermore, propofol has progressively replaced benzodiazepines [27,28,29], such as lorazepam, estrazolam, and midazolam [22,30,31], to reduce the primary adverse effects of these drugs for the mechanically ventilated sedation of patients in the intensive care unit (ICU) [32]. It can also prevent sleep apnea hypopnea syndrome (SAS) [33].

Like most drugs marketed and used, propofol possesses not only therapeutic effects but also unavoidable side effects [34,35]. From local side effects to propofol infusion syndrome [25,35], one of the most common adverse reactions of propofol is pain caused by local intravenous injection mediated by the activation of TRPA1 receptors on pain nerves [36]. Of course, this adverse reaction can be avoided by pre-injecting a local anesthetic. Additionally, other adverse reactions have emerged in clinical use, such as hypotension, respiratory depression, and cerebrovascular effects [37]. Even as certain adverse reactions show gradual improvement through combination therapy, the inherent structural limitations of the compound itself have increasingly drawn professional attention. This has spurred efforts to reformulate or restructure the compound, attempting to resolve issues at the route of administration or even at the structural root cause.

## 2. Trends in Propofol Analog Modification

Since propofol’s discovery, structural modifications have primarily focused on enhancing water solubility and mitigating adverse effects. Hydrogen bonding between propofol’s hydroxyl group and receptor molecules constitutes its primary binding mechanism [7]. Substituting the hydroxyl proton significantly reduces ligand-receptor affinity, with the effects of ortho-substituted hydrophobic groups appearing particularly pronounced.

Due to structural limitations inherent to propofol itself, the only functional group promoting water solubility is confined between two adjacent isopropyl groups, resulting in virtually zero water solubility and poor pharmacokinetics. Due to its extremely low oral bioavailability, it is primarily administered clinically as a fat emulsion. However, emulsion formulations injected into slow-flowing vessels (especially small veins) can cause significant local injection site pain and allergic reactions. Additionally, fat emulsions may induce lipid metabolism disorders [38,39,40], hyperlipidemia, and adverse effects like dyspepsia [37]. Other administration methods, such as nebulizer inhalation, mucosal delivery through upper gastrointestinal epithelial cells, or rectal suppository administration, have shown suboptimal clinical efficacy due to low bioavailability. Therefore, there is an urgent need for a method to directly enhance the water solubility of propofol.

Water-soluble prodrugs enhance the water solubility of propofol by forming pharmaceutical compositions, thereby enabling alternative administration routes beyond injection. These include enteral routes such as oral administration and other parenteral routes such as subcutaneous, mucosal, and inhalation delivery. Building upon the propofol structure, the design of water-soluble prodrugs primarily focuses on modifying the hydroxyl group. Introducing other water-soluble groups through acylation or phosphorylation of the oxygen atom facilitates formulation preparation and avoids the need for additional excipients like lipids, which could introduce extra risks.

Mitigation or avoidance of adverse reactions is addressed through two primary approaches: managing local adverse reactions and systemic adverse reactions. Local adverse reactions are significantly influenced by the route of administration and the inherent properties of the drug itself, and are primarily improved by enhancing formulation characteristics. Systemic adverse reactions, beyond those caused by excipients, fundamentally stem from inherent flaws in the compound itself. To reduce local injection pain and enhance the compound’s inherent safety, modifications were made to propofol’s structure by substituting the benzene ring and isopropyl substituents. The goal was to ensure equivalent efficacy at lower doses for the modified drug, thereby potentially increasing its safety profile. However, this approach does not simultaneously improve the compound’s solubility. Therefore, while designing structural analogs, solubility enhancement still relies on prodrugs.

## 3. Prodrug Strategy

### 3.1. Design of Water-Soluble Propofol Prodrugs

Modifications to the propofol hydroxyl group primarily involve the following structural features: carbonate or carboxylate esters, phosphates, amino or amino acid esters, and methoxy groups. Theoretically, any combination of two or more of these groups could provide additional hydrolysis sites, significantly enhancing water solubility without markedly increasing toxicity relative to the parent drug.

#### 3.1.1. Sodium Phosphopropofol Fospropofol

**Figure 2:**
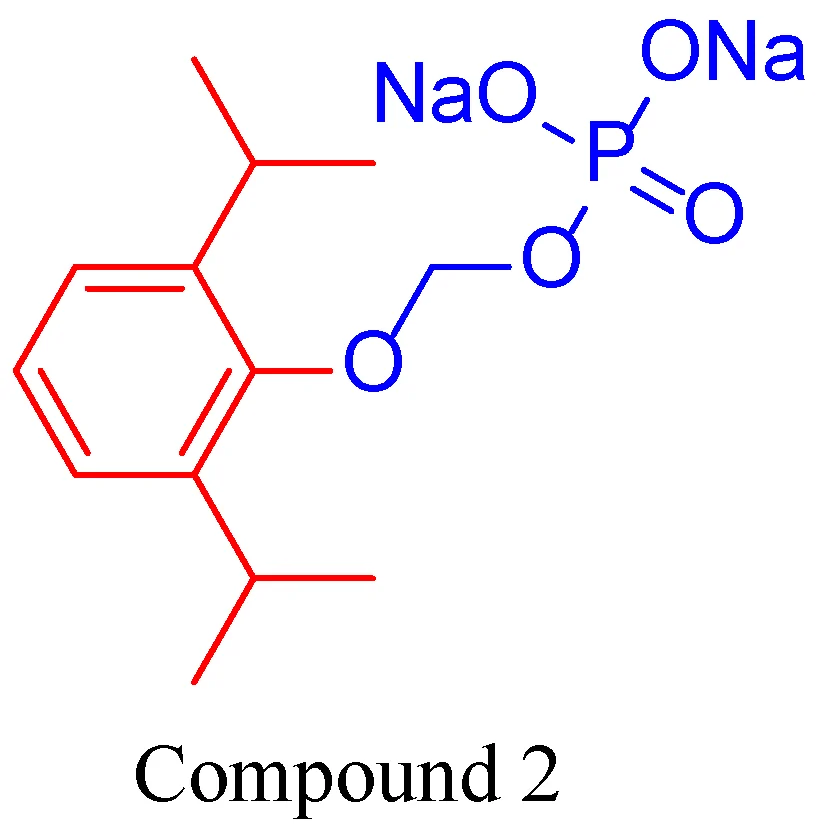
Chemical structure of sodium fospropofol.

Sodium fospropofol **(Compound 2)**, also known as GPI15751, is a phosphonoxymethyl ether-substituted 1-hydroxypropofol derivative, as shown in Figure 2. Developed by Eisai Inc. (Nutley, NJ, USA), it received FDA approval in December 2008 under the brand name Lusedra for intravenous administration [41,42].

A 2001 U.S. patent documented the drug’s synthesis method, and in vitro testing determined the water solubility of the propofol prodrug and its hydrolysis products [43]. Experiments demonstrated that this prodrug exhibits good stability at pH levels suitable for pharmaceutical formulation preparation but rapidly decomposes under physiological conditions in vivo, possessing a water solubility of approximately 500 mg·mL^−1^, hydrolyzing to propofol upon addition of alkaline phosphatase glycine buffer solution. The compound underwent systemic toxicity studies in rats and pharmacokinetic trials in dogs, collectively demonstrating the precursor drug’s safety and therapeutic consistency. In rats, inhibitory effects were observed within minutes after administration of high-dose (equivalent to 9 mg·kg^−1^ propofol) and medium-dose formulations, but doses sufficient to induce loss of righting reflex (LORR) were not reached. Recovery was complete, and no signs of adverse reactions attributable to the prodrug were detected. In beagle dog pharmacokinetic studies, compared with the propofol formulation Diprivan, equivalent doses of the propofol prodrug with a different formulation exhibited similar anesthetic recovery times (within 20–30 min) and blood concentrations.

In a 2003 report, sodium propofol was first tested in volunteers in preliminary clinical trials assessing safety, tolerability, pharmacodynamics, and pharmacokinetics. When three groups of three volunteers received constant-rate infusions of different doses of GPI15751 over ten minutes, no injection pain was reported. Only two volunteers experienced mild discomfort at the start of infusion. The infusion demonstrated good tolerability. At the high dose of 1160 mg, all volunteers in each group exhibited loss of consciousness at a plasma concentration of 2.1 μg·mL^−1^ ± 0.6 μg·mL^−1^. In comparison with propofol emulsion, based on the median effective dose (ED_50_), propofol achieves a concentration of 2.0 μg·mL^−1^ ± 0.5 μg·mL^−1^ at the site of action, whereas for propofol emulsion, the concentration was 3.0 μg·mL^−1^ ± 0.7 μg·mL^−1^, demonstrating improved efficacy at lower concentrations.

Experimental results indicate that such compounds possess favorable pharmacological effects. Combined with relevant content mentioned in other patents [44], these drugs can serve as sedatives and anesthetics, as well as treatments for migraines, epilepsy, pruritus, anxiety disorders, insomnia, nausea, and other medical conditions.

A randomized, double-blind, multicenter trial enrolled 127 patients requiring colonoscopy [45]. A midazolam group at 0.02 mg·mL^−1^ served as the positive control, while four experimental groups received propofol sodium at concentrations of 2, 5, 6.5, and 8 mg·mL^−1^. This study evaluated the safest and most effective dose of propofol sodium within specified concentrations following fentanyl premedication. As the dose increased, the sedative effect of propofol sodium continued to improve. However, all dose groups demonstrated good tolerability, with only four cases of sedation-related adverse events: two patients developed hypotension, and two patients experienced mild to moderate hypoxemia. There were no deaths or withdrawals. The 6.5 mg·mL^−1^ dose group demonstrated a balanced profile of sedation duration, depth, safety, and patient preference compared to both the lower-dose group and the 8 mg·mL^−1^ group.

Propofol sodium was classified as a controlled substance by the U.S. Drug Enforcement Administration (DEA) at the onset of its market availability. It must be administered by anesthesiologists trained in anesthesia, with strict regulations governing single-dose limits and patient age and physical condition. In clinical practice, the drug is metabolized in vivo to produce one molecule of formaldehyde, which has potential toxicity. When used in combination with other myocardial depressants or anesthetic analgesics, it may enhance myocardial depression [46].

#### 3.1.2. Other Phosphate and Carboxylate Prodrugs

**Figure 3:**
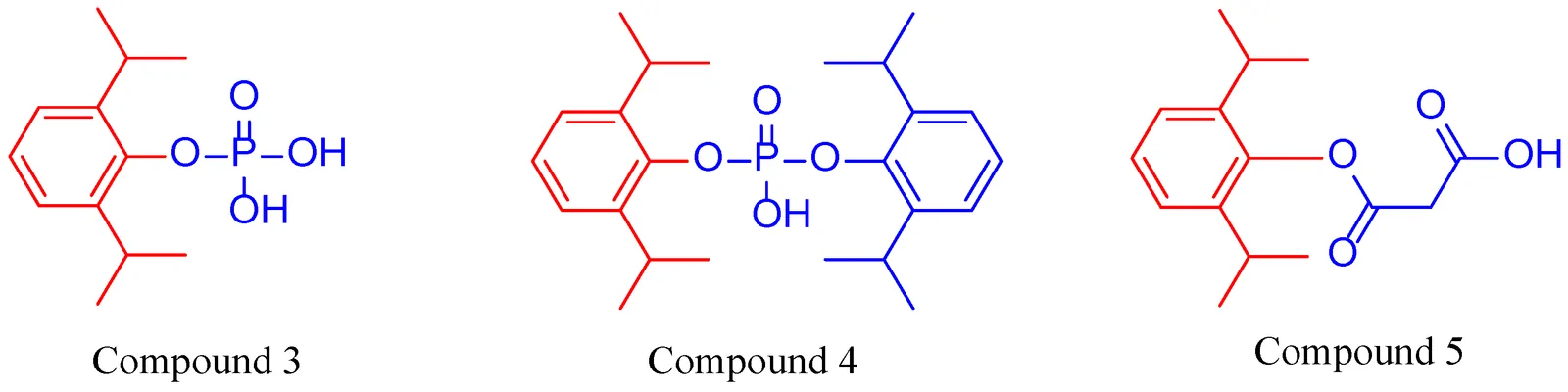
The pharmaceutical composition invented by V. J. Stella.

Propofol phosphate sodium, the first phosphate ester precursor drug, was synthesized in 1999 using a similar method to yield the directly linked propofol phosphate disodium salt or mono(propofol) phosphate **(Compound 3)**, propofol diphosphate **(Compound 4)**, and propofol hemisuccinate **(Compound 5)**, as shown in Figure 3 [44,47]. Compared to propofol, propofol hemisuccinate exhibits at least an order of magnitude higher solubility in water. In its in vivo hydrolysis studies, peak propofol concentrations in rats were observed 2–4 h post-administration. The average half-life of propofol phosphate hydrolyzed to propofol in human saliva is approximately four days. Initially, propofol’s faster recovery from anesthesia and reduced amnesia made it the most promising alternative to other sedatives, particularly for patients requiring only short-term sedation. However, for patients needing prolonged sedation, propofol’s utility requires continuous maintenance dosing to be achieved. Numerically, the water-soluble prodrug form of propofol diminishes the rapid onset and metabolism advantages inherent to propofol itself. Conversely, the reduced maintenance dosage appears applicable to a broader range of medical procedures.

**Figure 4:**
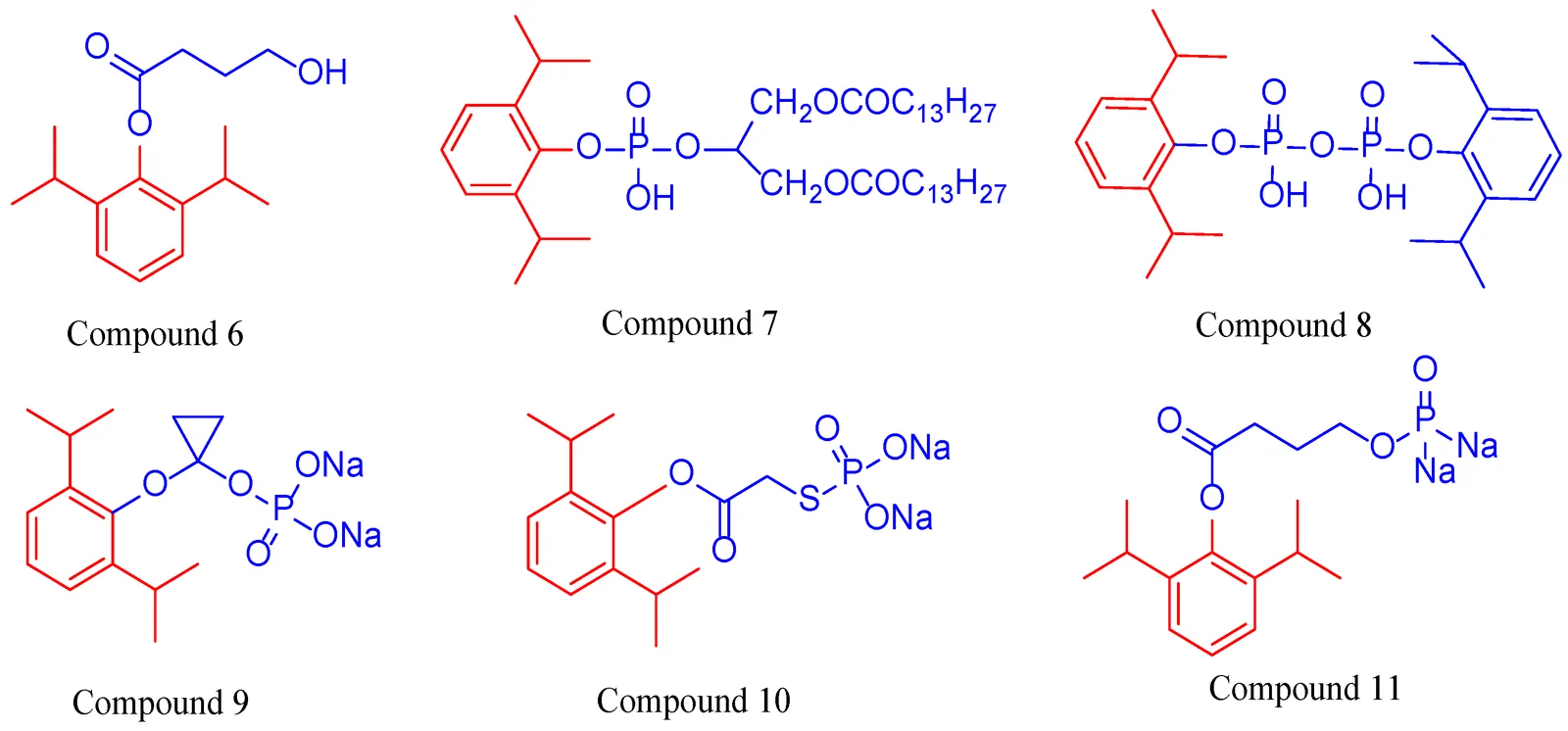
Structural design of phosphate prodrugs.

However, as proposed in the European patent application [48], although the sodium succinate monoester of propofol is a highly water-soluble prodrug, in vitro tests revealed its high instability, which is unfavorable for industrial production. Therefore, based on this, it was proposed to reduce the dicarboxylate by half, resulting in hydroxybutyrate succinate **(Compound 6)** (structure shown in Figure 4) and hydroxyvalerate succinate. These derivatives exhibit in vitro stability and rapidly hydrolyze into active propofol in plasma. In preliminary pharmacodynamic studies in mice, the ED_50_ of hydroxybutyrate succinate (52 mg/kg ± 35–67 mg/kg, 95% CI) was lower than that of the control agent Diprivan (6.1 mg/kg ± 5.1–7.9 mg/kg, 95% CI) in mice. The time to disappearance of the forelimb righting reflex (FORR, 45.3 s ± 12.3 s) and recovery time (235.6 s ± 67.9 s) were both slightly longer than those in the control group (onset time 19 s ± 3 s; recovery time 217.6 s ± 67.3 s), demonstrating stable onset and reversible anesthetic effects.

HX0969w [49,50] **(Compound 11)** is also a novel water-soluble propofol phosphate prodrug with an ED50 of 133.03 mg/kg in mice. Unlike sodium propofol phosphate, the structure of HX0969w comprises propofol, gamma-hydroxybutyrate (GHB), and a phosphate group, and it does not produce harmful formaldehyde molecules during metabolism in vivo.

Following the University of Kansas’s announcement of O-phosphonoxymethylpropofol derivatives, modifications to the specific phosphonic acid moiety of phosphonoxypropofol derivatives were reported in 2010, including saturated acyclic, cyclic, or heterocyclic groups and their combinations [51]. These groups were attached to the phosphorus atom via a linker containing one or more carbonyl or sulfonyl groups. The water solubility of **Compound 7** mentioned in the study (7.3 mg·mL^−1^) under neutral conditions at 25 °C is not representative. Moreover, due to sample size uncertainties, specific theoretical standards cannot be inferred from pharmacokinetic parameters. However, based on maximum plasma concentration, the half-life and plasma-time curve, this type of prodrug can indeed achieve a slow-release effect of propofol in animal models. Furthermore, increasing the chain length of the aliphatic group in the phosphonic acid moiety does affect the product’s solubility, which in turn impacts the preparation of oral formulations.

Guangzhou Institute of Chemistry, Chinese Academy of Sciences proposed the preparation of dipropofol pyrophosphate dihydrate (**Compound 8**) to address the metabolic issues of sodium phospho-propofol [52]. This compound links two propofol molecules via a phosphoric acid ester group, exhibiting water solubility of 27 mg·mL^−1^ at standard temperature and pressure. Notably, the onset time in both rats and mice was within minutes and shortened with increasing doses, while the duration of anesthesia gradually prolonged with higher doses. This structure demonstrates certain safety characteristics. Employing cycloalkane as the linker between the phosphate group and propofol residue ensures that the cycloalkanol generated upon in vivo degradation exhibits minimal toxic effects [53]. Compared to propofol, the eight compounds in the examples exhibited significantly shorter onset times for inducing anesthesia in mice, with the compound in Example 3 **(Compound 9)** demonstrating the shortest onset time. The duration of anesthesia was also markedly prolonged, with the compound in Example 16 exhibiting the longest duration. All differences were statistically significant.

Considering the patented method for preparing water-soluble propofol derivatives using phosphonoxy methyl as a synthetic building block, this approach struggles to achieve water-soluble prodrugs for drugs intolerant to high temperatures, resulting in low yields and high industrialization costs. A Chinese patent discloses phosphonyl carboxylic acid propofol ester derivatives, such as α-disodium phosphonomethylpropionate propofol ester **(Compound 10)**, along with its preparation method [54]. This method is claimed to feature mild reaction conditions, high yields, straightforward operation and industrialization potential. Following comparison with commercially available propofol formulations via rat tail vein injection, the oral formulation derived from this derivative demonstrated high bioavailability, rapid absorption, and excellent stability. Due to the derivative’s excellent water solubility, it can be formulated as an injectable solution. This eliminates the need for adjuvants like surfactants in the formulation, which pose safety concerns. On one hand, this enhances formulation stability; on the other, it reduces or even eliminates injection pain, improving patient compliance. Not only does it overcome the shortcomings of propofol emulsions, it also offers the added benefit of faster onset of action.

#### 3.1.3. Amino Acid Substituent Analogs Amino

Acid substituents represent another area of significant focus in propofol formulations. These amino acids may be linear or cyclic. Among propofol’s amino acid derivatives, those bearing amino groups constitute a highly effective class of metabolically homologous molecules within the body, ensuring unquestionable safety. The amino group can serve not only as the terminal free radical of the prodrug, forming carbonates, carboxylate esters, or phosphates, but also as a bridging substituent forming part of a metabolic bridge. This allows it to combine with carboxyl, phosphate, and methoxy groups to create complex oral carrier delivery scaffolds. This approach was subsequently adopted in the modification of benzene-ring alkyl substituents.

Cyclic amino acid derivatives [55]. The water solubility of L-proline salt 6a **(Compound 12)** and phthalate salt 6c is 350 mg·mL^−1^ and 525 mg·mL^−1^, respectively, representing a thousandfold increase compared to propofol’s solubility (0.15 mg·mL^−1^) under identical conditions. The IC_50_ value of **Compound 12** in physiological saline solution is one order of magnitude higher than that of propofol (4.17 μM). The team conducted in vitro receptor binding assays for the series of compounds. Combined with voltage-clamp electrophysiology measurements on African clawed frog oocytes, GABA-induced chloride currents in cloned GABAA receptors were concentration-dependently enhanced by 6a and attenuated by 6d, reaching maximum effects at 50 and 100 μM, respectively. L-proline salt modulated the GABAA receptor with intrinsic activity, though this activity was lower than that of propofol. In vivo comparative studies via intraperitoneal injection in rats demonstrated that L-proline salt, acting as an in vitro agonist, exhibited optimal prodrug properties for anticonvulsant activity. Similar to propofol, it provided complete protection against pentylenetetrazole-induced seizures.

The anesthetic potency of this prodrug was investigated by measuring the onset of loss of righting reflex (LORR) and duration in mice, and compared with the clinical propofol formulation Diprivan and an oil/water mixture of Tween 80. Following intraperitoneal administration of the propofol water-in-oil emulsion, the induction time to loss of righting reflex (114.4 s ± 9.5 s) was significantly shorter than that observed visually with the Diprivan formulation (289 s ± 14.8 s). The induction time for the L-proline saline solution (162.7 ± 4.3 s) fell between that of the water-in-oil formulation and Diprivan, while the duration of anesthesia followed the pattern: propofol water-in-oil emulsion (2245 s ± 252 s) < L-proline (2403 s ± 592 s) ≈ Diprivan (3895 s ± 111.3 s). Thus, L-proline exhibits the advantages expected of a short-acting anesthetic, with a duration of action comparable to Diprivan but a significantly shorter induction time.

**Figure 5:**
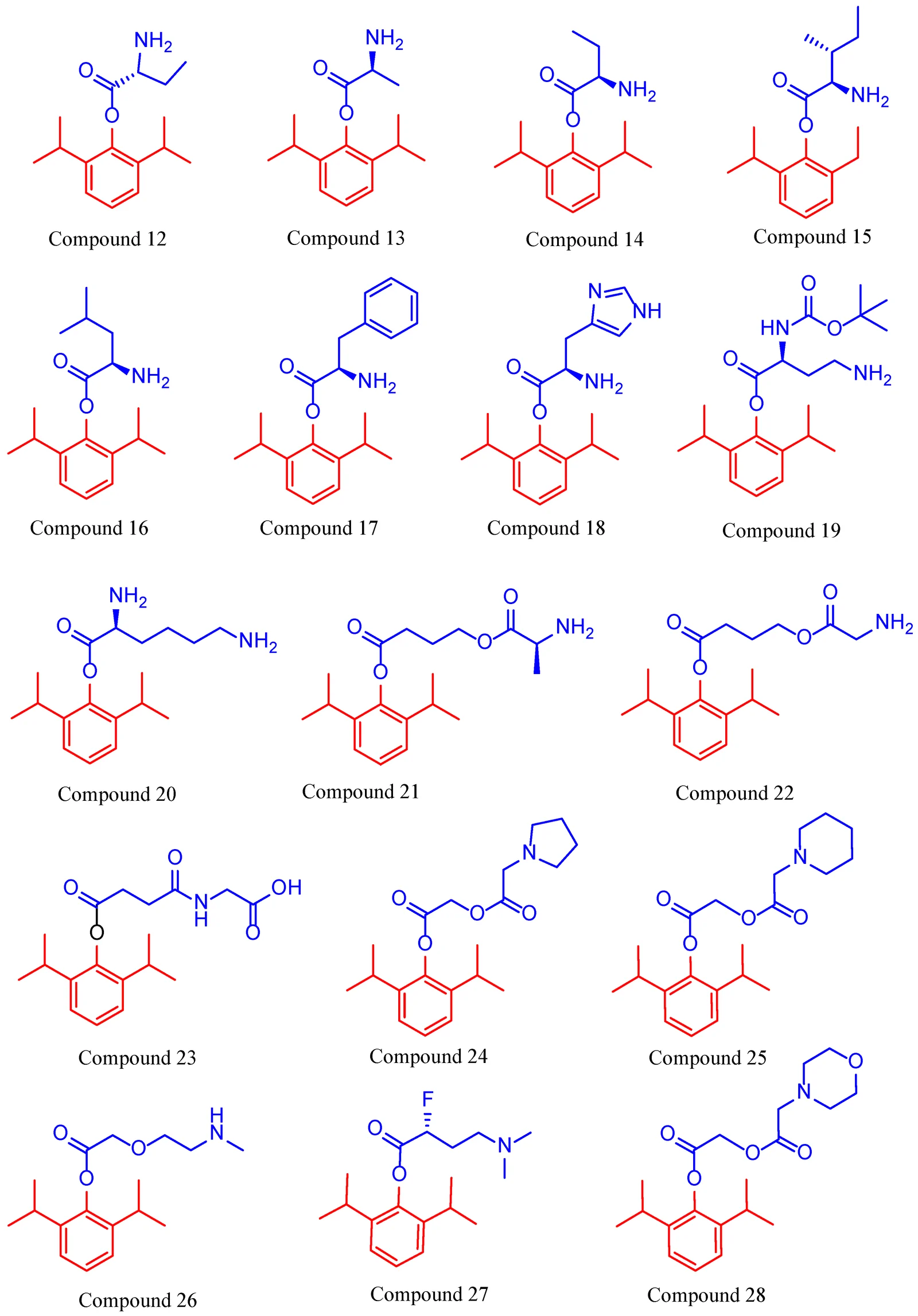
Cyclic amino acid esters and human amino acid ester analogs.

The Chinese team also investigated esters substituted with different amino acids [56] (Table 1), including essential amino acids such as alanine (**Compound 13**), valine (**Compound 14**), isoleucine (**Compound 15**), leucine (**Compound 16**), and phenylalanine (**Compound 17** and **Compounds I1–I12**), as shown in Figure 5. All target compounds were evaluated for water solubility and stability, achieving double-digit solubility values. Their half-effective dose in aqueous solution was comparable to that of Diprivan, positioning them as highly promising prodrug 13 candidates. Basic amino acid ester salts include histidine (**Compound 18**), lysine (**Compound 20**), and ornithine (**Compound 19**) [57]. Their water solubility at standard temperature and pressure exceeds that of propofol by a thousandfold. When stored at room temperature in the dark, histidine hydrochloride, arginine hydrochloride, and lysine hydrochloride exhibit excellent stability in water with notable pharmacological effects. Their onset time decreases in a dose-dependent manner, while recovery time from the righting reflex shows the opposite trend. Similar to initial studies, propofol succinate was modified by adding glycine or alanine at the terminal end [58], yielding propofol hydroxybutyrate glycine or alanine hydrochloride or acetate salts. These rapidly degrade in vivo. Using mouse LORR as an endpoint, their aqueous solution onset times approached those of Diprivan, with longer duration and a median effective dose (ED_50_) twice as high. Another similar patent reported the water solubility of propofol succinate monoester glycine amide (**Compound 23**) sodium salt to be greater than 250 mg·mL^−1^ [59]. In animal safety evaluations (acute and chronic toxicity studies), the median lethal dose (LD_50_) values were 53 mg/kg–55 mg/kg for mice and 42 mg/kg–45 mg/kg for rats. In rats administered daily infusions of 10 mg·kg^−1^–30 mg·kg^−1^ for 30 days, no toxicity occurred and histopathological examination revealed no significant pathological changes.

A major drawback of some amino acid ester formulations is their tendency to cause drug accumulation. At effective doses, the recovery time following administration is longer than with direct propofol use. These factors can delay postanesthesia recovery, increase monitoring costs, and pose additional postoperative risks to patients.

Jiangsu Enhua Luokang Pharmaceutical Research Co., Ltd. (Xuzhou, China) investigated the effects of different nitrogen substituents and configurations in amino acid fluorocarboxylates on their degradation rates and pharmacodynamics in rabbit or mouse plasma [60]. Observations indicate that direct fluorination at the α-carbon atom increases degradation rates in animals, with the R configuration further enhancing this effect. Conversely, more complex substituents on the nitrogen atom hinder rapid plasma degradation. Among these, **Compound 27**, structurally represented as propofol 4-(N,N-dimethyl)amino-2-(R)-fluorobutyric acid ester hydrochloride in physiological saline solution, exhibited the following parameters at the same concentration (3 mg·mL^−1^) in mice: LD_50_, ED_50_, and therapeutic index were 71.8, 17.9, and 4.3, respectively. Compared to the commercially available propofol lipid emulsion injection (38.4 mg·mL^−1^, 7.9 mg·mL^−1^, and 4.9 mg·mL^−1^), the therapeutic window is narrower, yet it still exhibits good anesthetic efficacy, rapid onset, and short duration.

Chongqing Medisun Pharmaceutical Technology Co., Ltd. (Chongqing, China) introduced an oxygen atom between the amino and carboxylate groups to develop a class of water-soluble prodrugs for propofol or its analogs that exhibit rapid onset (rapid in vivo release of alkyl-substituted phenol) and swift recovery post-dosing (minimal drug accumulation) [61]. Compared to diprivan and sodium propofol in physiological saline solution, the designed **Compound 26** exhibited shorter onset times (0.2 ± 0.05 min) for anesthesia in rats, as well as shorter recovery times for righting reflex (22.4 ± 5.2 min) and spontaneous activity (29.8 ± 13.2 min) after discontinuation. It more closely resembled propofol than propofol sodium. This indicates that while addressing water solubility, the compound better retains propofol’s advantages of rapid onset and short duration of action. Additionally, no respiratory arrest or animal deaths were observed in the compound group, demonstrating superior safety.

Insertion between propofol and cyclic amino acids accelerates release of the prodrug [62]. **Compound 24**, **Compound 25**, and **Compound 28** released all active propofol into the bloodstream within one minute in physiological saline solution, compared to propofol sodium. Animal studies demonstrated that this prodrug significantly outperformed propofol phosphate in safety, onset time, and duration of anesthesia. Its molar dose, onset time, and duration of anesthesia were comparable to propofol, preserving the clinical advantages of propofol while exhibiting a relatively higher therapeutic index.

**Table 1:** Pharmacodynamic Characteristics in Mice. a: Time from the end of intravenous injection to the onset of loss of right-side recumbency (LORR); b: time from the onset of LORR to recovery from LORR.

Compound	ED50/mg/kg	Loss of the Righting Reflex, LORR	
		Onset/s ^a^	Duration/min ^b^
Propofol [62]	11.7 ± 1.2	1.0 ± 0.7	4.6 ± 0.7
Fospropofol [62]	105.0 ± 10.5	46.4 ± 6.0	41.2 ± 13.2
6a [55]	EC50 = 2–10 μmol·mL^−1^	162.7 ± 4.3	40.05 ± 9.8
Human amino ester derivatives (I 1–12) [56]	9.85–13.53	-	-
L-alanine ester hydrochloride [58]	28.3	60.6	31.4
Glycine ester hydrochloride [58]	19.6	150	49.3
L-alanine ester trifluoroacetate [58]	25.8	12	31.6
Glycine ester triacetate [58]	21.1	12	49.3
Propofol succinate monoester glycine amide sodium salt [59]	87.66–167.32	936 ± 612	15.6 ± 10.2
**Compound 28** [62]	28.1 ± 2.7	3.1 ± 1.8	5.5 ± 0.9
**Compound 24** [62]	25.2 ± 2.5	4.0 ± 0.8	7.3 ± 4.6
**Compound 25** [62]	22.3 ± 2.0	3.0 ± 0.8	6.4 ± 1.7

(a) time from the end of injection to the appearance of LORR; (b) time from the appearance of LORR to the recovery of LORR.

#### 3.1.4. Double-Effect Anesthetic Composition and Polyethylene Glycol-Conjugated Prodrug

**Figure 6:**
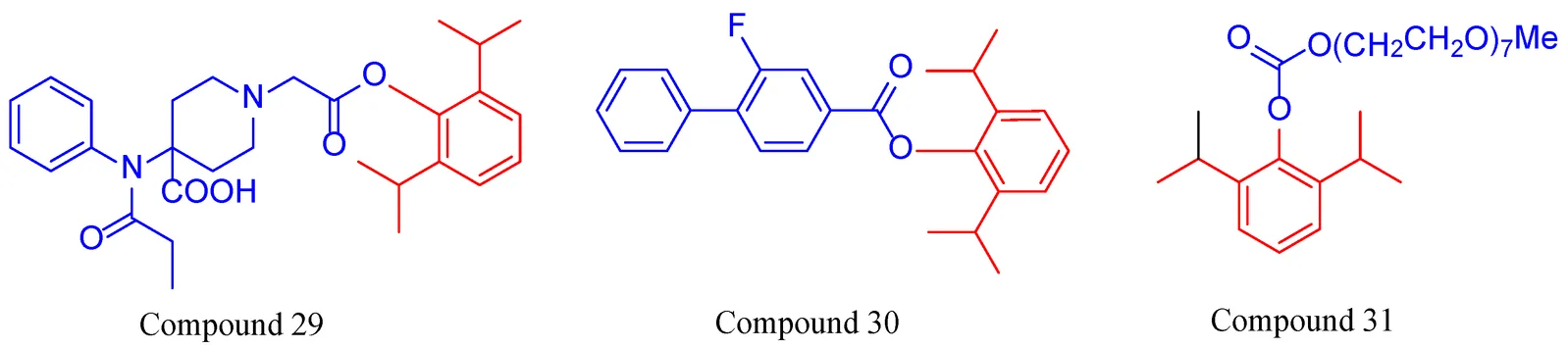
Double-effect composition and polyethylene glycol-conjugated prodrug.

There are other methods for preparing prodrugs, such as dual-molecule dual-effect anesthetic compounds and polyethylene glycol-conjugated prodrugs (Figure 6). The U.S. FDA has approved several synthetic polymers for intravenous administration, among which polyethylene glycol-drug conjugate and polyethylene glycol-drug conjugate (PEG-DRUG) modification techniques have seen continuous development in recent years for use in injectable delivery systems. Following activation, PEG links to drug molecules. Upon entering the body, it is slowly hydrolyzed to release the small drug molecules for therapeutic effect. This approach offers advantages, including increased drug water solubility, reduced 18 toxicity, improved pharmacokinetics, and enhanced drug stability. PEG-propofol has also been reported to exhibit these advantages. The monodisperse polyethylene glycol monomethyl ether-modified propofol prodrug **(Compound 31)** demonstrated increased water solubility with longer PEG chains, exhibiting anesthetic effects within safe therapeutic doses without toxic side effects, with a duration of action under ten minutes [63]. Single-arm, double-arm, and quadruple-arm PEG-propofol derivatives exhibited good photostability and thermal stability [64]. Their effective propofol-equivalent doses were comparable to Diprivan, but mice showed longer times to loss and recovery of righting reflex than with Diprivan, indicating a longer half-life. This may increase the risk of adverse anesthetic effects and monitoring costs.

The design of bimolecular drugs primarily stems from integrated considerations for postoperative analgesia and intraoperative sedation, combining sedatives and anesthetics into a single agent to provide a novel anesthetic drug with both analgesic and anesthetic effects. Clinically, anesthesiologists must continuously adjust dosages based on patient needs and pain levels. Separately administering continuous infusion or target-controlled infusion of propofol and analgesics to maintain effective drug concentrations significantly increases operational complexity. Numerous historical anesthesiology publications have documented the combined use of propofol with remifentanil and flurbiprofen. Guangdong Jiabo Pharmaceutical Co., Ltd. (Qingyuan, China) and West China Hospital of Sichuan University independently proposed prodrug combinations for these drug pairs [65,66]; however, further research was not pursued after efficacy was demonstrated.

### 3.2. Tertiary Amine Salt Derivative

**Figure 7:**
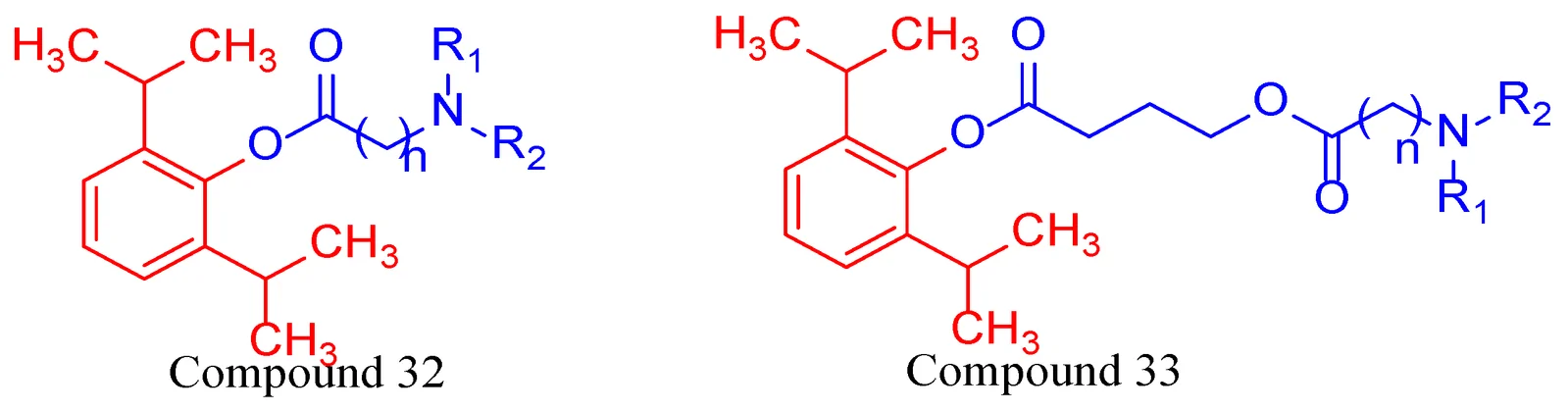
The structures of the series of amine salt derivatives.

Existing propofol derivatives primarily enhance solubility by introducing hydrophilic groups such as phosphate, polyethylene glycol, or amino groups. However, these modifications often suffer from limitations, including the release of harmful substances during enzymatic conversion, slow in vivo drug release, and instability in aqueous solutions. To address these shortcomings, researchers have conducted extensive studies on propofol structural modifications. In 2024, teams from Zhengzhou University and the Chinese Academy of Medical Sciences published research in Bioorganic & Medicinal Chemistry Letters [67]. Using propofol as the lead compound, they innovatively designed two classes of tertiary amine salt derivatives, with the core difference lying in the structural optimization of the connecting arms:

**I.** Series Derivatives **(Compound 32)** (structure shown in Figure 7)**:** Employing the structural motif “propofol + α-aminoacetic acid ester/β-aminopropionic acid ester,” with acetyl or propionyl groups serving as connecting arms, 14 target compounds (**I-1** to **I-14**) were synthesized via a three-step reaction, as shown in Table 2. The synthesis began by reacting propofol with chloroacetyl chloride or bromopropionyl chloride to form intermediates. These were then subjected to N-alkylation reactions with various amines, followed by salt formation to yield the 24 target products. This design focused on enhancing water solubility via the amino ester moiety.

**II.** Series of Derivatives **(Compound 33)**: The innovative “dual-functional connecting arm” design was introduced to establish the structural feature of “propofol + γ-hydroxybutyric acid + α-aminoacetic acid ester/γ-aminobutyric acid ester.” Twenty compounds (**II-1** to **II-20**) were synthesized through a five-step reaction sequence. The synthesis process first generates an intermediate via the acylation reaction of propofol with succinic anhydride. Following sodium borohydride reduction, sequential acylation, amination, and salt formation reactions are performed. Among these, γ-hydroxybutyric acid exhibits neuroinhibitory effects, producing synergistic anesthetic effects with propofol, while the aminoacetic ester group enhances water solubility, achieving the dual objectives of “solubilization + synergistic efficacy.” The following are the structural formulas of the two series of derivatives.

ED_50_: The dose of a drug that produces a positive response (effective response) in 50% of experimental animals or subjects under specific experimental conditions. It is a key indicator for evaluating drug potency in dose–response relationships; a lower ED_50_ indicates higher drug potency. LD_50_: The dose of a drug that causes 50% of experimental animals to die under specific experimental conditions. Using KM mice as a model, the study determined the median effective dose (ED_50_), median lethal dose (LD_50_), onset time, and duration of anesthesia for the derivatives via tail vein injection. Representative compounds were selected for in vitro drug release experiments in mouse plasma. Key findings are as follows: all 34 target compounds retained moderate anesthetic potency with significantly enhanced safety profiles: LD_50_ values ranged from 3.9 to 62.8 mmol/kg (compared to propofol at 1.6 ± 0.3 mmol/kg). Twenty-nine compounds exhibited a therapeutic index (TI) exceeding that of propofol (TI = 2.7), with Series II derivatives demonstrating particularly outstanding performance. All Series II compounds had a TI greater than 4.8, significantly higher than the 1.4–4.5 range observed in Series I compounds. Regarding onset speed, the target compounds exhibited an average onset time of 14.6 s, comparable to propofol (15 s), demonstrating rapid onset advantages. Anesthetic duration was significantly prolonged, ranging from 137 to 571 s, with Series II compounds achieving 3–10 times the duration of propofol. This addresses propofol’s short duration of action and the need for repeated dosing.

#### 3.2.1. Performance Advantages of Optimized Compounds

Compound **II-20** from the II series demonstrated optimal comprehensive performance: a TI of 5.6 (twice that of propofol), an onset time of only 11 s, and an anesthetic duration of up to 571 s (ten times that of propofol). With no significant in vivo toxicity, it emerged as a highly promising candidate drug. Additionally, compounds **II-10** and **II-17** from the II series also exhibit outstanding properties, with TI values of 5.1, onset times of 16 s and 8 s, respectively, and durations of 547 s and 413 s, providing diverse options for different clinical scenarios.

#### 3.2.2. In Vitro Drug Release Characteristics

Five representative compounds—**I-2, I-11, II-8, II-10**, and **II-20**—were selected for HPLC analysis. Results demonstrated rapid propofol release in mouse plasma for all compounds: 71–94% drug release within 20 min. By 120 min, II series compounds achieved 96% release, significantly higher than the 80% observed for the I series. This difference stems from the smaller steric hindrance of the II series compounds, facilitating easier hydrolysis in plasma. This ensures the rapid establishment and maintenance of effective drug concentrations in vivo, consistent with in vivo anesthetic activity data. Based on experimental results, the study hypothesizes that the performance differences between the two derivative classes are closely related to their mechanisms of action [68]: One hydrolysis product of Series II compounds is γ-hydroxybutyric acid (GHB), which possesses intrinsic neuroinhibitory effects and acts synergistically with propofol; it synergistically enhances central inhibitory effects with propofol, prolonging anesthetic duration. Simultaneously, the reduced steric hindrance in Series II enhances plasma enzyme hydrolysis efficiency, accelerating propofol release for rapid onset.

From a structure–activity relationship perspective, the design of the connecting arm is pivotal in determining derivative performance; the introduction of a bifunctional γ-hydroxybutyric acid connecting arm not only enhances water solubility and safety but also synergistically optimizes anesthetic potency and duration. Moreover, the magnitude of steric hindrance directly regulates the drug release rate, thereby influencing the onset time and duration of action. This discovery provides crucial guidance for subsequent propofol derivative design: by rationally selecting linker types and modulating steric hindrance, precise optimization of anesthetic potency, safety, and release characteristics can be achieved [69].

## 4. Active Structural Analog Strategy

### 4.1. Substitution and Modification of Substituents on the Phenyl Ring of Propofol

Deuterated halides [70] were used to replace hydrogen atoms on the benzene ring to confirm that cleavage of the C–H bond on the aromatic ring does not affect propofol’s anesthetic properties [71]. While the ortho position of the phenol group was occupied by isopropyl, the para and meta positions remained unsubstituted.

#### 4.1.1. Para-Substitution on the Benzene Ring

**Figure 8:**
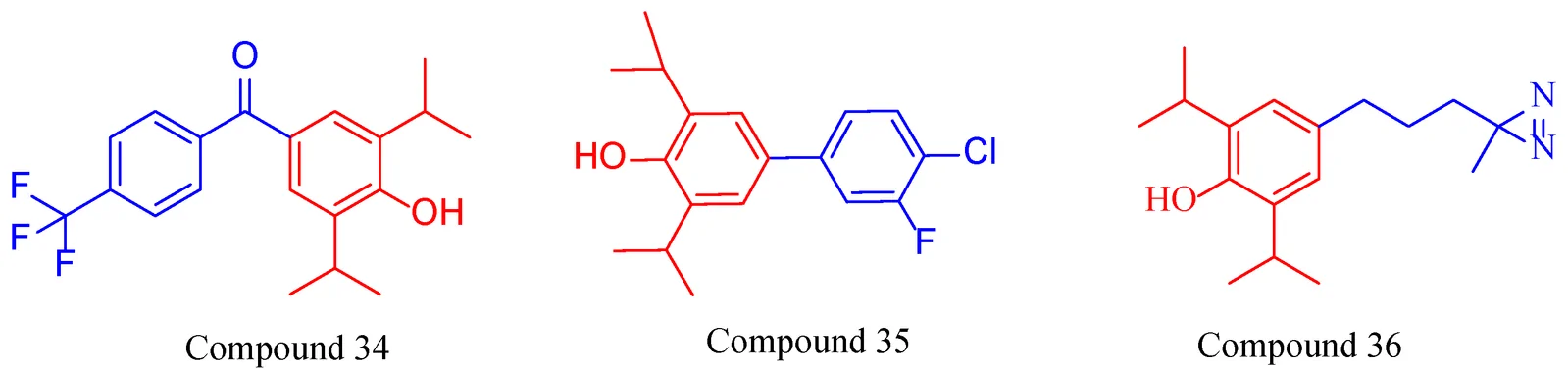
Typical para-substituted analogs.

A benzene ring or heteroaromatic ring was attached to the para-position of the phenol group via a carbonyl linkage, with the expectation that these new compounds would exhibit greater activity in inducing anesthetic effects. It was anticipated that such compounds could be used in high doses for general anesthesia while inducing hypnotic, sedative, and sleep-inducing effects at lower doses, thereby reducing propofol-associated side effects (e.g., cardiovascular adverse effects). However, compounds like CT8 **(Compound 34)** (structure shown in Figure 8) [71] have lost clinical value as anesthetics due to poor penetration through peripheral tissues. Subsequently, some analgesics deliberately designed with para-substitution, such as p-(3-fluoro,4-chlorophenyl)-propofol (**Compound 35**) [72], gradually deviated from anesthetic purposes, acting as glycine receptor agonists rather than GABAA receptor agonists. P-(4-azipentyl) propofol, also known as p-4-AziC5-Pro (**Compound 36**) [73], exhibits anesthetic potential similar to propofol. However, it has only been reported as a photoreactive probe to aid in identifying and characterizing residues involved in the propofol binding site, with no further studies on its clinical therapeutic effects.

#### 4.1.2. Benzocyclobutene Derivatives

The meta-position of the hydroxyl group was found to provide favorable therapeutic indices and sufficient potency when substituted with a methyl group. Moreover, numerous drug molecules containing a cyclobutane moiety—such as ivabradine (**Compound 37**) [74] for treating heart failure and sibutramine (**Compound 38**) [75] with appetite-suppressing and antidepressant activities—demonstrate that the cyclobutane moiety possesses sufficient in vivo stability. This allows for a reduction in the molecular weight by 20% in the isopropylphenol molecule, enhancing molecular rigidity and providing a chiral benzyl center for various substituents. Consequently, a series of novel benzocyclobutene derivatives exhibited potent anesthetic effects and superior therapeutic indices compared to propofol.

The research team at Haisco Pharmaceutical Group first observed that methyl substitution at the meta position of the isoflurane phenol ring enhanced the therapeutic index. Consequently, they fused a 2-isopropyl group to the meta position, proposing a phenyl-propylcyclobutene skeleton [76]. Early pharmacodynamic validation of this structure rapidly confirmed its significant potential, demonstrating that **Compound 1a (Compound 39)** exhibited a therapeutic index twice that of propofol (TI = 5.4 vs. TI = 2.7), comparable median effective dose (16.0 mg·kg^−1^ vs. 11.7 mg·kg^−1^), onset within 10 s, and duration exceeding 300 s. It exhibited favorable pharmacokinetics in rats, with a half-life (38 ± 14 min) comparable to propofol. **Compound 40**, derived from compound 1a by substituting a hydrogen atom of the benzyl group with a cyano group, exhibits a higher therapeutic index (TI = 9.1) and potent efficacy (ED_50_ = 8.2 mg·kg^−1^). Further structure–activity relationship (SAR) studies indicated that small hydrogen bond acceptor (HBA) groups, such as cyano, are effective for good ED_50_ and a wider therapeutic range. However, subsequent evaluation of the indazole-based structures revealed that these analogs, which mimic the phenolic hydroxyl group with an aniline moiety, did not exhibit sufficient activity. This indicates that the phenolic hydroxyl group is essential for maintaining good activity, and therefore, the intramolecular interaction may not be the primary factor contributing to the improved potency and safety profile [73].

#### 4.1.3. Ciprofol

**Figure 9:**
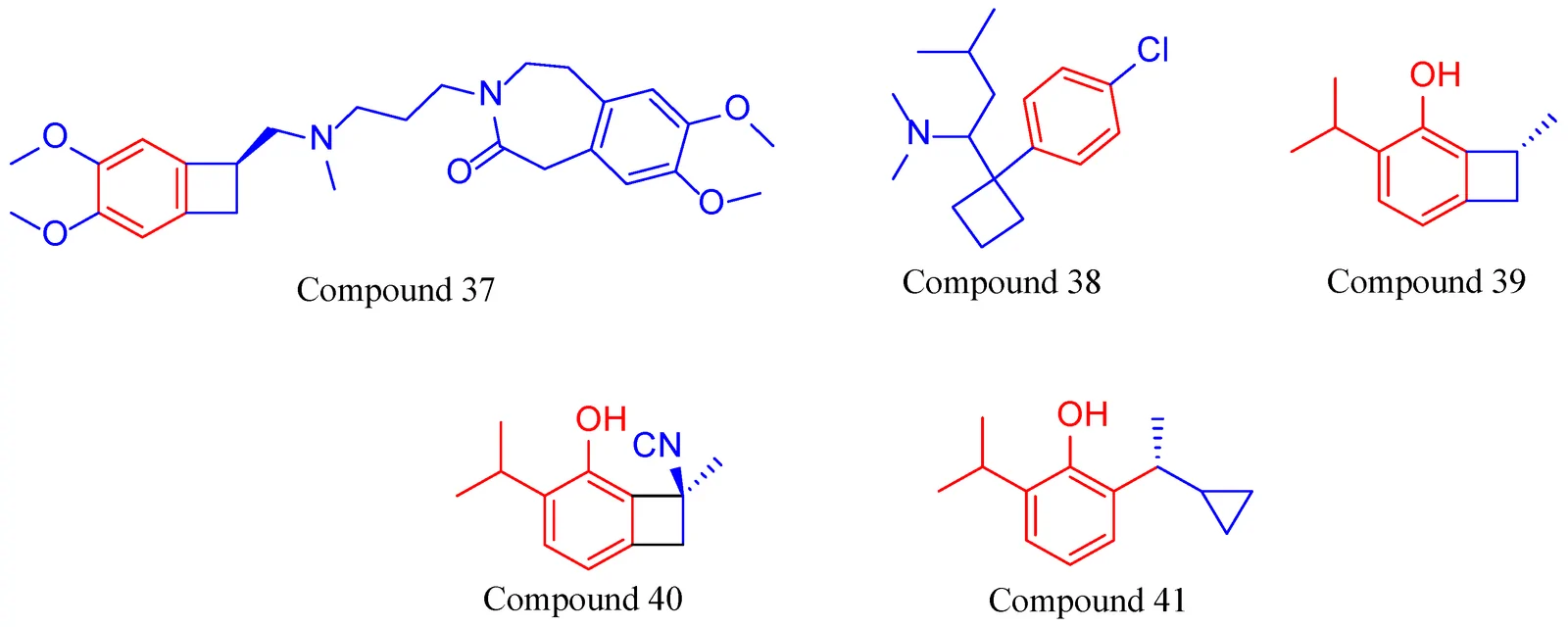
Drugs with a cyclobutane structure and drugs developed by Haisco.

The Anesthesiology team at West China Hospital developed cyclopropane propofol-**HSK3486 (Compound 41)** (structure shown in Figure 9) [77] and subsequently applied for patent authorization in over 20 countries, including China (including Hong Kong), the United States, Australia, Europe, Canada, Japan, and South Africa. The study compared several groups of compounds with differing lipophilicity and steric bulk. Compared to propofol, the R-configuration Ciprofol incorporates a cyclopropyl group on the isopropyl chain, disrupting the unique symmetry of the propofol molecule. This not only introduces increased steric effects but also provides a chiral carbon atom to satisfy the stereoselectivity required for anesthetic efficacy, resulting in superior anesthetic performance compared to propofol. Its low-dose onset facilitates the formulation of lower-concentration emulsions. Compared to fospropofol, the phosphate salt of Ciprofol exhibits equivalent sedative effects while demonstrating significantly shorter onset and recovery times. Its effective dose (ED_50_ = 1.5 mg·kg^−1^) is also markedly lower—one-fifth that of fospropofol.

Ciprofol is formulated as an emulsion injection, representing a novel intravenous anesthetic with proprietary intellectual property rights. It is intended for multiple indications, including colonoscopy sedation, induction of anesthesia, maintenance of anesthesia, and ICU sedation. To date, Haisco Pharmaceutical Group has conducted 14 Phase I clinical studies, 6 Phase II clinical studies, and 6 Phase III clinical studies following its development. Data from the Phase II clinical trial for the “colonoscopy sedation” indication were presented at the American Society of Anesthesiologists Annual Meeting. Compared to propofol, ciprofol demonstrated superior efficacy at equivalent doses (0.4 mg·kg^−1^–0.5 mg·kg^−1^ vs. 2 mg·kg^−1^), with comparable induction (1.2 min) and recovery times (5.5 min). In safety assessments, it demonstrated a lower incidence of injection pain (9.5% vs. 45.2%), reduced potential for respiratory depression (11.1% vs. 16.1%), and lower lipid consumption, thereby alleviating metabolic burden—particularly beneficial for patients with hepatic dysfunction or severe illness during maintenance. Currently, Haisco Pharmaceutical Group is conducting a Phase III clinical trial comparing Ciprofol with propofol for efficacy and safety in inducing general anesthesia, marking the product’s expansion into the new indication of “general anesthesia maintenance.” 

Ciprofol is an independently developed Class I new drug, competing with domestic counterparts such as Hengrui’s remimazolam tosylate and Renfu’s remimazolam benzenesulfonate. Based on current clinical trial data, it demonstrates “me-better” potential relative to the originator drug propofol.

#### 4.1.4. Other Isopropyl-Modified Analogs

**Figure 10:**
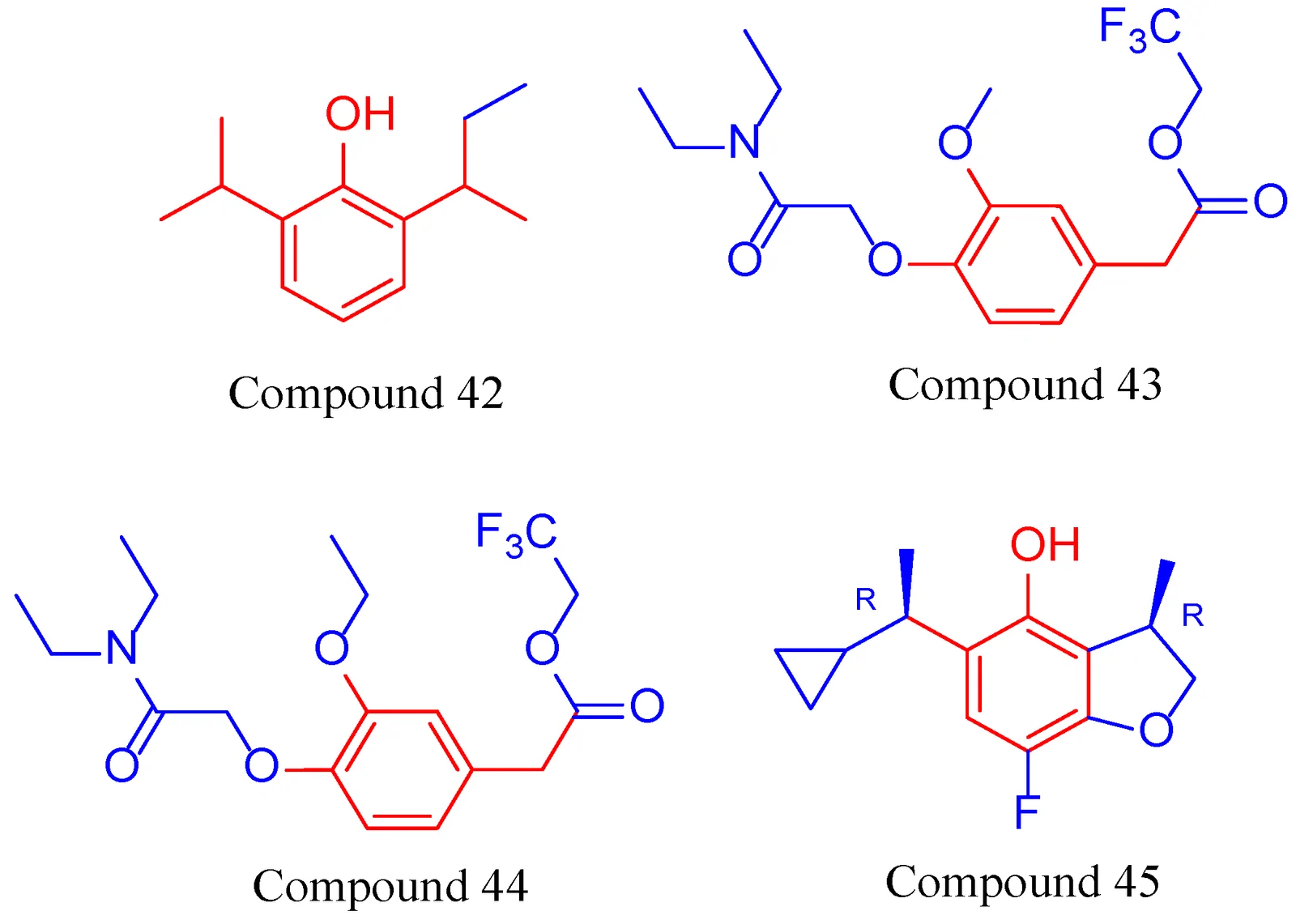
Other asymmetric analogs.

Early reports hinted at the immense potential for propofol modification offered by this asymmetry and chirality [70]. The Jenkins Thomas team designed numerous analogous compounds. Compared to propofol, **Compound 42** achieved modification by attaching a methyl group to the isopropyl chain, demonstrating improved safety, enhanced hemodynamic properties, and good anesthetic efficacy. Experimental studies established models, including the righting reflex rat, anesthetized pig, and Beagle dog to evaluate the compounds’ pharmacokinetic characteristics and anesthetic performance. These demonstrated optimization against adverse effects such as hypotension and blood gas abnormalities, equivalent plasma clearance, higher potency at lower doses, a higher maximum tolerated dose, and an increased therapeutic index. Intravenous access was established in beagle dogs to monitor anesthetic depth, revealing anesthetic onset and duration comparable to propofol. **Compound 43** [68] and **Compound 44** [68] (structure shown in Figure 10) exhibited shorter induction periods at effective doses compared to propofol (8.5 ± 1.9 s) and propanidid (6.5 ± 1.3 s), at 6.2 ± 1.2 s and 5.3 ± 0.5 s, respectively; and a shorter duration of anesthesia compared to propofol (166 ± 155 s) and propanidid (73 ± 20 s), at 44.8 ± 3.5 s and 60.1 ± 8.4 s, respectively. These modifications to the isopropyl asymmetry indicate promising prospects, but the substitution of the propofol hydroxyl group further impairs its water solubility.

Building on the exploration of asymmetric isopropyl modifications, the researchers further proposed a novel conformational restriction strategy. Based on this strategy, a dihydrobenzofuran-based propofol analog, 53A **(Compound 45)**, was designed and synthesized: by cyclizing one of the alkyl substituents of propofol to construct a [5,6]-fused bicyclic scaffold, the number of rotatable bonds in the molecule was reduced, which may be beneficial for blood–brain barrier permeability. The scaffold was divided into three regions, A, B, and C, wherein an R^1^ group was introduced at the 4-position of region A to inhibit phenolic oxidation, and a variety of hydrophobic substituents R^2^/R^3^ were systematically investigated in regions B/C to screen for the optimal candidate compound. In the preliminary structure–activity relationship exploration, the 3-dimethylated analog exhibited significantly reduced activity, whereas increasing the steric bulk of the substituent by introducing a propyl group restored the activity, with the cyclopropyl derivative being the most potent. Accordingly, it was hypothesized that the superior properties of the optimal compound may arise from a combination of multiple factors: the conformational constraint reducing rotatable bonds, the cyclopropyl group enhancing hydrophobic interactions, and the introduction of fluorine both strengthening hydrogen bonding and blocking metabolic sites. Single-crystal X-ray diffraction (SCXRD) analysis confirmed the absolute configuration of 53A to be (R,R). In the loss of righting reflex (LORR) assay, the ED_50_ of 53A was approximately 4.00 mg/kg; compared with propofol, 53A was approximately fourfold more potent, while also exhibiting a shorter onset time and a longer duration of anesthesia. Furthermore, 53A displayed a wider safety margin: the therapeutic index (TI) in mice was 6.172 versus 5.061, and in rats it was 4.362 versus 2.580. Respiratory safety assessment showed that the respiratory depression induced by 53A was milder in intensity than that of propofol, with the depression being immediately alleviated upon recovery (approximately 10 min after injection), whereas the reduction in respiratory rate induced by propofol persisted; 53A also restored the minute ventilation to the level of the saline control within 10 min after administration. When administered as a lipid emulsion, the rat LD_50_ of 53A was approximately twice that of the solution formulation, with a corresponding increase in the therapeutic index, and these changes were even more pronounced with propofol. Overall, 53A exhibited a superior therapeutic index compared with propofol and a faster recovery from anesthesia, making it a promising candidate for clinical development [78].

### 4.2. Silylpropofol

**Figure 11:**
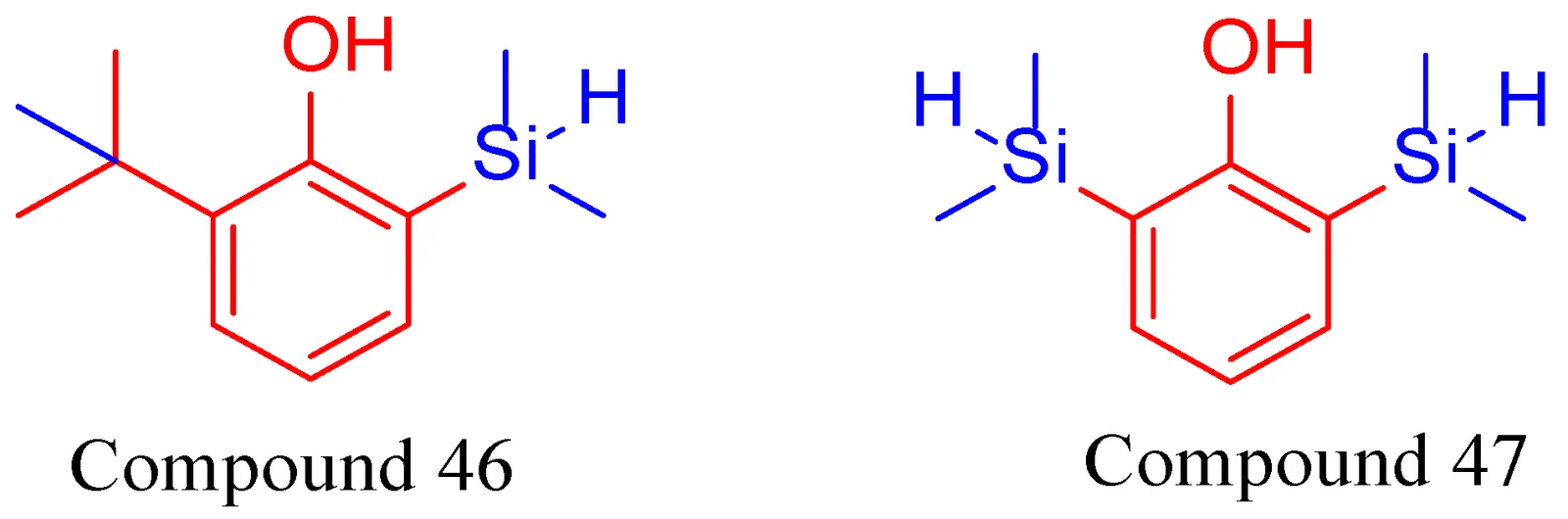
Ailane derivatives: mono-silapropofol and di-silapropofol.

A 2026 study published in ACS Omega innovatively applied a carbon-silicon isosteric strategy to propofol structural modification, successfully synthesizing the first silylated propofol analogs—mono-silylated **(Compound 46)** and di-silylated propofol **(Compound 47)** (structure shown in Figure 11) [79]. This breakthrough offers a novel approach to anesthetic drug design and lays a crucial foundation for elucidating propofol’s mechanism of action. Carbon-Silicon Isomerization Strategy (also known as carbon-silicon conversion, CSS) serves as a potent tool in medicinal chemistry for modulating molecular properties. Given the similarity in valence electron structures between silicon and carbon, CSS leverages differences in atomic radius (silicon being larger) and electronegativity (silicon being less electronegative than carbon) to alter bond polarity, polarizability, and electron distribution. This preserves the overall spatial conformation while influencing molecular solubility, metabolic stability, and receptor binding characteristics. Previous applications of this strategy in structural optimization of drugs like ibuprofen and haloperidol have demonstrated its potential for enhancing biological activity and elucidating mechanisms of action. Addressing propofol’s structural limitations and mechanism research needs, this study proposes a core design approach: replacing one or both isopropyl groups at the ortho-position of propofol’s benzene ring with dimethylsilyl groups. This yields mono-silapropofol **(Compound 46)** and di-silylated propofol **(Compound 47)**. Research objectives include: establishing efficient synthetic routes to obtain high-purity target compounds; elucidating the effects of carbon-silicon substitution on molecular structure and electronic properties through crystal structure analysis and quantum chemical calculations; and evaluating the aqueous solution stability of silyl analogs under physiological conditions to provide data supporting their potential as anesthetic drugs or tools for mechanism studies.

Stability under physiological conditions is a critical indicator for drug application. This study compared the hydrolysis stability of mono-silapropofol **(Compound 46)** and di-silylated propofol **(Compound 47)** in simulated physiological environments using ^1^H NMR monitoring: in neutral physiological saline (0.9% NaCl/D_2_O), mono-silapropofol **(Compound 46)** exhibited progressive hydrolysis: purity decreased from an initial 98% to 78% after 2 weeks, and nearly complete hydrolysis occurred after 6 weeks. Hydrolysis products are presumed to include 2-isopropylphenol, dimethylsilanol, and other unknown byproducts. Under weakly alkaline conditions (10 mM NaHCO_3_/D_2_O, pH ≈ 8), the hydrolysis rate significantly accelerated, with only 60% of the target compound remaining after 4 days, indicating poor stability in alkaline environments. Di-silylated propofol **(Compound 47)** exhibited markedly superior stability: no significant degradation was observed in neutral physiological saline over 6 weeks, demonstrating excellent aqueous stability. However, hydrolysis still occurred under weakly alkaline conditions, with 80% remaining after 4 days and only 30% after 2 weeks, suggesting an adverse effect of alkaline environments on the stability of the silicon-carbon bond. The differing stabilities of silylated propofol derivatives offer diverse application directions: di-silapropofol’s high stability in neutral physiological environments enhances its potential as a novel anesthetic candidate. The increased polarity from carbon-silicon substitution may improve propofol’s water solubility, reducing formulation challenges and injection site pain. Mono-silapropofol’s progressive hydrolysis characteristics make it suitable as a probe molecule for investigating propofol’s metabolic pathways and receptor binding kinetics [67]. Furthermore, the unique electronic properties and electrostatic potential distribution of both compounds enable their use as tool molecules to elucidate the binding mechanism between propofol and GABAA receptors, providing new targets for structural optimization of anesthetic drugs.

## 5. Summary

Propofol, as a well-established anesthetic agent, is classified as a generic drug in the World Health Organization’s Essential Medicines List. Its intravenous formulation appears milky, earning it the affectionate nickname “milk” among anesthesiologists. Beyond causing injection site pain and lipid metabolism disorders, it is also known as “amnesia milk” due to its tendency to suppress memory recall. Since replacing thiopental sodium, it has become nearly indispensable for general anesthesia. Later, with the introduction of aliphatic propofol, its impact remained limited due to dosage constraints and restricted applications. However, thanks to research by numerous teams, promising analogs continue to emerge—HSK3486 **(Compound 39)** being a prime example. These compounds hold potential to compete with propofol, and propofol derivative development has evolved from ‘simple solubilization’ toward multidimensional strategies, among which the combination of solubilization and synergistic pharmacodynamics represents one of the emerging research directions in the field. “Structures featuring low steric hindrance and dual-functional linkers” represent a core direction for future water-soluble prodrug design. A groundbreaking study published in ACSO mega in 2026 overcame the limitations of traditional propofol derivatives, which relied solely on functional group modifications to improve physicochemical properties. Through atom-level isosteric substitution, it introduced an innovative approach for anesthetic drug design: “spatial conformation retention coupled with electronic property regulation.”

From a clinical perspective, addressing injection pain is a critical goal for next-generation propofol formulations. An ideal pain-free formulation should possess three key characteristics. First, it should exhibit extremely high water solubility, allowing direct preparation as an aqueous solution without relying on lipid or surfactant excipients, thereby fundamentally reducing or eliminating injection pain and avoiding risks such as lipid metabolism disorders. For instance, fospropofol achieves a water solubility of approximately 500 mg·mL^−1^, and early volunteer trials have reported no injection pain during constant-rate infusions. Second, the prodrug must demonstrate high chemical stability during formulation and storage to support industrial production; however, some highly water-soluble prodrugs, such as propofol hemisuccinate, exhibit significant instability in vitro, which is unfavorable for industrial production. Third, the prodrug must undergo rapid and complete release of propofol upon entering the body to ensure rapid onset and stable maintenance of anesthesia. This water-soluble prodrug strategy, characterized by high solubility, formulation stability, and rapid in vivo release, represents the fundamental direction for solving injection pain and related adverse reactions associated with propofol emulsions.

## Figures and Tables

**Table 2:** Anesthetic activities of a synthesized series of amine salt derivatives.

Compound	*n*	NR1R2	ED50 (mmol/kg)	LD50 (mmol/kg)
**I-1**	1	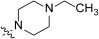	8.0 ± 0.3	11.5 ± 0.4
**I-2**	1	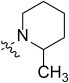	9.2 ± 0.5	15.8 ± 0.4
**I-3**	1	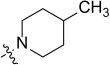	11.1 ± 0.4	18 ± 0.8
**I-4**	1	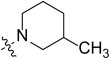	1.7 ± 0.1	3.9 ± 0.1
**I-5**	2	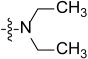	17.0 ± 0.3	58.8 ± 0.9
**I-6**	2	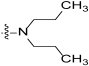	15.2 ± 0.2	57.6 ± 0.4
**I-7**	2	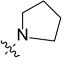	15.1 ± 0.1	3.9 ± 0.1
**I-8**	2	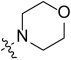	12.0 ± 0.5	43.8 ± 0.2
**I-9**	2	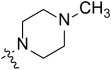	9.0 ± 0.1	33.4 ± 0.4
**I-10**	2	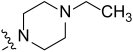	12.0 ± 0.1	38.0 ± 0.13
**I-11**	2	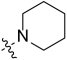	8.2 ± 1.0	36.9 ± 0.62
**I-12**	2	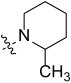	14.0 ± 0.7	57.7 ± 0.36
**I-13**	2	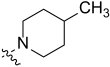	9.2 ± 0.3	38.9 ± 1.67
**I-14**	2	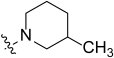	7.1 ± 0.6	28.7 ± 0.30
**II-1**	1	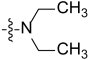	11.0 ± 0.2	52.8 ± 0.3
**II-2**	1	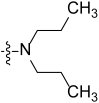	9.0 ± 0.6	45.9 ± 8.1
**II-3**	1	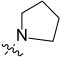	11.3 ± 1.5	56.5 ± 1.8
**II-4**	1	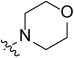	8.2 ± 0.3	39.6 ± 0.3
**II-5**	1	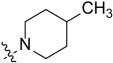	3.6 ± 0.1	17.6 ± 0.7
**II-6**	1	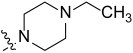	10.1 ± 0.7	50.5 ± 0.3
**II-7**	1	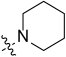	8.3 ± 0.2	39.8 ± 0.6
**II-8**	1	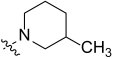	13.0 ± 0.5	62.8 ± 6.1
**II-9**	1	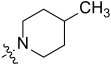	8.6 ± 0.3	40.6 ± 3.7
**II-10**	1	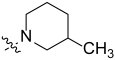	2.9 ± 0.1	14.7 ± 0.1
**II-11**	3	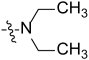	7.1 ± 0.5	36.3 ± 1.3
**II-12**	3	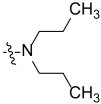	9.2 ± 0.7	48.1 ± 3.7
**II-13**	3	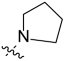	8.9 ± 0.3	46.4 ± 2.1
**II-14**	3	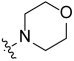	3.6 ± 0.7	17.5 ± 0.7
**II-15**	3	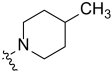	4.1 ± 0.6	21.4 ± 1.9
**II-16**	3	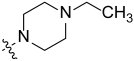	6.5 ± 2.5	31.9 ± 2.1
**II-17**	3	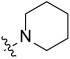	2.4 ± 0.1	12.3 ± 1.3
**II-18**	3	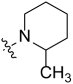	6.2 ± 0.4	32.1 ± 0.6
**II-19**	3	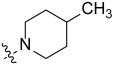	2.5 ± 0.1	13.7 ± 0.1
**II-20**	3	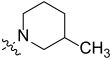	1.9 ± 0.1	10.7 ± 1.5

## Data Availability

No new data were created or analyzed in this study. Data sharing is not applicable to this article.
